# The Management of Children in Sickness and in Health

**Published:** 1880-06

**Authors:** 


THE MANAGEMENT OF CHILDREN IN SICKNESS AND IN HEALTH—A Book for Mothers. By Amie M. Hale, M. D. Presley Blakiston, Philadelphia, 1880. Price, 50 cents.

This little book of one hundred and odd pages has been forwarded to us through Messrs J. J. & S. P. Richards. It abounds in much sound and useful advice in reference to the management of children in health and disease. Not only may nurses and mothers be benefited by the suggestions contained in it, but physicians, too, will find much instruction and profit in reading its pages.

We commend it to the careful perusal of all who may be called upon to exercise the principle it announces.

